# Influence of physicochemical parameters on storage stability: Microbiological quality of fresh unpasteurized fruit juices

**DOI:** 10.1002/fsn3.500

**Published:** 2017-08-19

**Authors:** Phoebe P. Kaddumukasa, Samuel M. Imathiu, Julius M. Mathara, Jesca L. Nakavuma

**Affiliations:** ^1^ Department of Food Technology Faculty of Science Kyambogo University Kampala Uganda; ^2^ Department of Food Science and Technology Faculty of Agriculture Jomo Kenyatta University of Agriculture and Technology Nairobi Kenya; ^3^ College of Veterinary Medicine Animal Resources and Biosecurity Makerere University Kampala Uganda

**Keywords:** Bacteria, juices, unpasteurized

## Abstract

Fresh juices rich in health and nutritional benefits are valued for their fresh flavor, taste, and aroma. These juices' quality however is affected by factors like temperature, light, and microbiological contamination significantly changing physicochemical parameters and storage stability. Physicochemical and microbiological analyses of passion fruit, pineapple, and mango juices in dark and light bottles at 24°C and 4°C were conducted in Kampala, Uganda for 12 days. Physicochemical parameters significantly reduced (*p* < .05) storage stability of fresh juices, while no significant changes (*p* > .05) were observed for the microbiological analyses. pH values ranged from 3.0 to 4.2 (dark) bottles and 2.9 to 4.0 (light) bottles for juices at 24°C and 4°C. °Brix values were from 1.0 to 5.5 for dark and clear bottles at 24°C and 4°C. TTA (%) values ranged from 1.1 to 7.2 (dark) bottles and 1.1 to 7.4 for (light) bottles at 24°C and 4°C. Ascorbic acid content ranged from 3.5 to 61.0 mg/ 100 ml and 5.5 to 56.7 mg/100 ml for juices in dark and clear bottles, respectively. total plate counts ranged from 1.3 × 10___ to 3.3 × 10^7^ CFU/ml (dark bottles at 24°C) to 3.5 × 10³ to 3.3 × 10^8^ CFU/ml (dark bottles at 4°C). For juices in light bottles, total plate counts ranged from 1.8 × 10___ to 8.0 × 10^7^ CFU/ml (24°C) and 2.7 × 10___ to 1.5 × 10^8^ CFU/ml (4°C). High microbial loads suggest the use of poor processing techniques and lack of good hygiene which lower quality and reduce storage stability of juices. Storage temperature greatly reduces physicochemical parameters both at ambient and refrigeration temperatures. This implies that temperature control for unpasteurized juices is critical in order to inhibit microorganism metabolic activities which accelerate biodeterioration leading to spoilage and short shelf life. Fresh unpasteurized juices stored at 24°C and 4°C may safely be consumed within 1 and 2 days, respectively.

## 1 INTRODUCTION

Fresh unpasteurized juices are aqueous liquids obtained from fruit or vegetable tissue usually extracted manually or mechanically from these raw materials (Nma & Ola, 2013; Bello, Bello Temitope, Fashola Muibat, & Afolabi, 2014). Juices in the human diet offer a number of benefits including antioxidants, fiber, phytonutrients, vitamins, and minerals useful in preventing disease (Serpen, 2012; Tortoe, Johnson, Slaghek, Miedema, & Timmermans, 2013; Lamport, Saunders, Butler, & Spencer, 2014). Because of these nutritional benefits, consumers have developed a habit of daily consumption of fresh fruits and vegetables or their juices which have positive impact on health (Bhat & Stamminger, 2014; Simforian, Nonga, & Ndabikunze, 2015). Expressed juice may be consumed immediately or stored under refrigeration for later use, although, their shelf life under refrigeration has been reported to be short (De Corrêa Souza, Cristina, Benassi, & de Almeida, 2004).

Fruits and vegetables that have been minimally processed have unprotected skin and cell walls, and the fluid components are easily contaminated by air and microorganisms from the environment (Abbo, Olurin, & Odeyemi, 2006). Freshly expressed juice made from fruits and vegetables is highly susceptible to spoilage leading to deterioration of organoleptic and physicochemical parameters causing rejection of the product by consumers. Juice preparation involves a lot of human manipulation which has been reported to introduce microorganisms to the product (Redmond & Griffith, 2009; Artes & Allende, 2012). The microorganisms may be categorized as pathogenic or spoilage ones and the sources may include healthy food handlers (Redmond & Griffith, 2009). Food handlers have transient bacteria such as, *Staphylococcus aureus* resident on their hair, skin, throats, and nasal passages (Centre for food safety fruits for sale or serving in retail outlets, 2006). Bacteria find their way into the juice during processing when no protective apparel is used during food handling (Mihajlovic, Dixon, Couture, & Farber, 2013). Bacteria are also introduced into food products due to the frequent immersion of hands in water resulting in soreness and damage to the skin causing wounds (Centre for Food Safety, 2006). Wounds are good sites for bacterial pathogens especially when food handlers use bare hands. When food handlers prepare juices with bare hands, these pathogens come in contact with the juice. Bacterial transfer is facilitated through physical contact when wet hands are used during food preparation (Redmond & Griffith, 2009). Microorganisms are also introduced through direct contact with animal or human waste or indirectly with contaminated water, soil, processing equipment which can lead to spoilage of the juice (Mihajlovic et al., 2013). For economic reasons, water used in the washing of processing equipment and utensils may be recycled, further contaminating the product resulting into spoilage (Muyanja, Nayiga, Brenda, & Nasinyama, 2011). Juice spoilage leads to deterioration of organoleptic and physicochemical parameters causing rejection of the product by consumers leading to financial losses by the processor. On a more serious note, contamination may also be a potential microbiological health hazard to the consumer. Physicochemical parameters including storage temperature, pH, chemical composition, total soluble solids, color, and ascorbic acid also influence biodeterioration of fruit juices (Abbo et al., 2006; Nayik, Tawheed, & Sumanvikas, 2013). High temperatures allow the proliferation and growth of microorganisms in food products resulting into increased metabolic reactions, deterioration, and spoilage of the products leading to reduced storage stability or short shelf life. Food products are stored under refrigeration (0–4°C) to discourage the proliferation of bacterial cells, germination of spores, and possible toxin production to potentially dangerous levels (Redmond & Griffith, 2009). Increase in pH from 6 to 7 is optimum for the growth of mesophilic bacteria. Increased numbers of bacteria lead to the accumulation of metabolic by‐products leading to biodeterioration of juices and possibly spoilage. Vital nutrients such as antioxidants, viz. vitamins A, C, E, and phytonutrients are also destroyed due to exposure to light and temperature fluctuations during storage which results in reduced shelf life of the product. Decreased shelf life due to deteriorative reactions such as microbial spoilage, development of off‐flavors, change in color, texture, or appearance leads to degradation of the product, making it unacceptable to consumers (Amiri Sedigheh, 2008). Consumers in universities, offices, schools, shops, markets, roadside stalls, motorists, and even travelers may fall victim to contaminated juice resulting in foodborne illnesses. In addition, vendors do not adhere to good manufacturing practices and some lack proper personal hygiene during juice processing. High microbial numbers in fresh unpasteurized juices from unhygienic processing techniques increase consumers' exposure to health hazards. Changes in temperatures alter physicochemical parameters resulting in compromised shelf life of the juices. This study, therefore sought to establish the influence of physicochemical parameters (pH, Brix, titratable acidity, and ascorbic acid) on storage stability and microbiological quality of fresh unpasteurized fruit juices kept at ambient (24°C) and refrigeration (4°C) temperatures.

## 2 MATERIALS and METHODS

### 2.1 Fresh unpasteurized fruit juice

Fresh juice was obtained from three specific fruit varieties which included purple passion fruit (*Passiflora edulis*), pineapple, smooth cayenne (*Ananas comosus*), and mango, Ngowe (*Mangifera indica*). The fruits are readily available in the market and largely used by vendors in juice processing.

### 2.2 Physicochemical analyses of fresh unpasteurized fruit juices

Juices for physicochemical analyses were extracted in the laboratory as follows; blending of the fruit pulp using an electric blender (Kenwood, England) was done after proper washing under running tap water. This was followed by filtering to remove the seeds and the fibrous material. Juice was then diluted with isovolumes of water. The diluted juice sample was divided into four portions of equal volume (ca.500 ml) and kept either in a clear (light) bottle or a dark‐colored (amber) glass bottle. Isovolumes of juice samples in sterile clear (light) and dark (amber)‐colored bottles were placed in an ice box and immediately transported to the microbiology laboratory for analysis. Both types of bottle (clear and dark) with juice were stored; one at ambient (24°C) temperature and the other at refrigeration (4°C) temperature for each fruit juice type. The temperatures were selected because they were used at the retail outlets, so they were chosen for comparison of the findings.

Analyses including total soluble solids, pH and titratable acidity were determined at the start of the experiment and throughout the storage period for 12 days (Bello et al., 2014). Total soluble solids (TSS) were determined using an ATAGO refractometer (0–32°) and expressed as °Brix. pH of each juice sample was measured using a digital pH meter (Nig 333, Naina Solaris Ltd, India). Titratable acidity was measured according to Bello et al. (2014) and expressed as % citric acid. Ascorbic acid was determined and expressed in mg/ml according to AOAC method 972.1 (AOAC, 2002).

### 2.3 Microbiological analysis of fresh unpasteurized juices

Total plate counts were conducted using the standard plate method (Morton, 2001). Twenty‐five millimeters of juice sample was mixed with 225 ml of sterile buffered peptone water. A 10‐fold serial dilution for each sample was made using sterile buffered peptone water up to 10^−^___ dilution. Dilutions of 10^−^___ and 10^−^___ were surface spread onto duplicate sterile plate count agar (PCA) (Oxoid Ltd, Basingstoke, Hants, England) and incubated at 37°C for 24 hr, after which colony‐forming units were counted. Total plate counts were repeated every 48 hr during the period of storage.

### 2.4 Statistical analysis

One‐way ANOVA for total plate counts followed by the use of the *t*‐test for comparison of means of physicochemical parameters was performed using Graphpad prism version 7.00 for Windows, Graphpad software, La Jolia California, USA, www.Graphpad.com.

## 3 RESULTS and DISCUSSION

### 3.1 Physicochemical analyses of fresh unpasteurized juices

**Figure 1:**
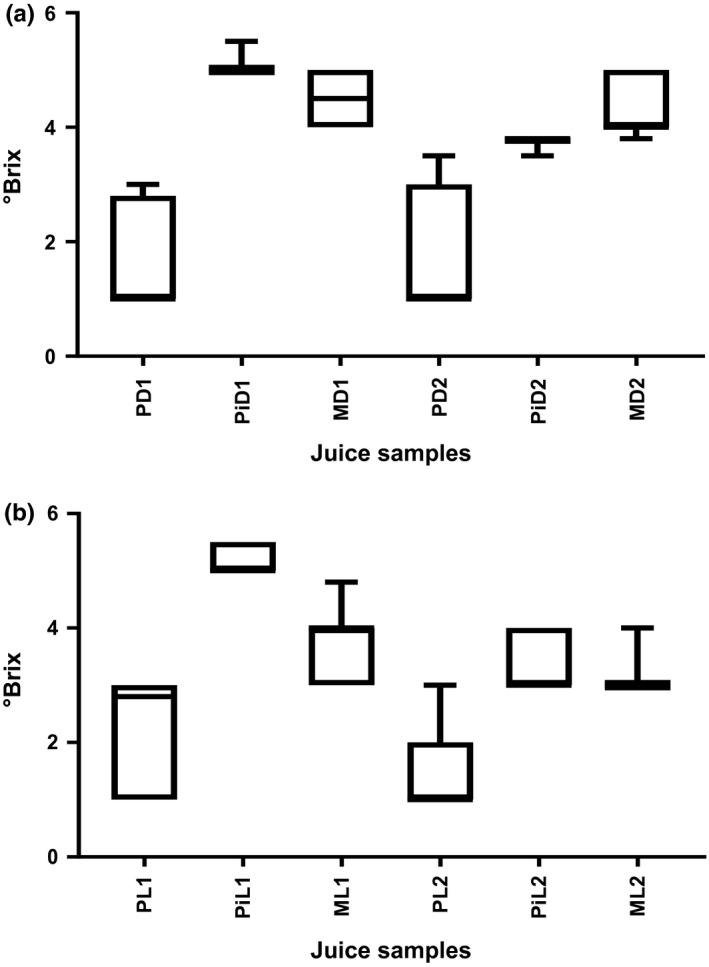
Mean °Brix levels of fruit juices kept in dark (amber)‐colored and clear (light) bottles at ambient (24°C) and refrigeration (4°C) temperatures. P‐Passion fruit, Pi‐ Pineapple, M‐mango, D‐ Dark bottle, L‐Light (clear) bottle, 1‐Ambient (24°C) temperature, 2‐ refrigeration (4°C) temperature

On average, a general decline in °Brix values was observed for juice samples stored at ambient (24°C) temperature (Figure 1). Values ranged from 5.5 to 1.0 for juices stored in both dark (amber)‐colored bottles and clear (light) bottles at ambient (24°C) and refrigeration (4°C) temperatures. °Brix values reported in the study were lower than values of 12, 11–12.8, and 11–16 for passion fruit (*Passiflora edulis*), pineapple (*Ananas comosus*), and mango (*Mangifera indica*), respectively, reported by Codex Alimentarius, (2005). This could possibly be due to the absence of any additives and fermentation of the available sugars in the juices to ethyl alcohol, carbondioxide, and water at ambient (24°C) temperature.

Specifically, °Brix values for juice samples in this study, ranged as follows: 3–1 for passion fruit, 5.5–5.0 for pineapple, 5–4 for mango; and 3.5–1 for passion fruit, 3.8–3.5 for pineapple, and 4.5–3.8 for mango juices kept in dark bottles at ambient (24°C) and at refrigeration (4°C) temperatures (Figure 1a). Passion fruit and mango juice samples stores at ambient (24°C) temperature did not significantly change (*p* > .05) from samples kept at refrigeration (4°C) temperature. However, °Brix values for pineapple stored at ambient temperature were significantly different (*p* < .05) from samples stored at refrigeration temperature. The decline may probably have been due to microbial metabolic activities resulting in the conversion of the sugars in the samples to organic acids leading to reduction in pH, spoilage, and shortened shelf life. A similar trend was observed for juices stored in clear (light) glass bottles.

Silva, da Silva, & Abud, 2015 reported °Brix values of 11.2, 12.5, and 10.3 for passion fruit, pineapple, and mango pulps, respectively, values of which were higher than those obtained in the study. Brix values in this study were low possibly due to dilution of the prepared juices. Secondly, low Brix values could also probably be due to the variation in seasons of harvest and levels in fruit ripeness. Vendors purchase gardens/fields of fruits and vegetables well ahead of time before they reach the ripe stage to meet market demand. In this way some of the fruits may not be fully developed resulting in juices with low physicochemical attributes. These fruits are covered in heaps to induce ripening which probably influences some of the attributes like the °Brix levels.

**Figure 2:**
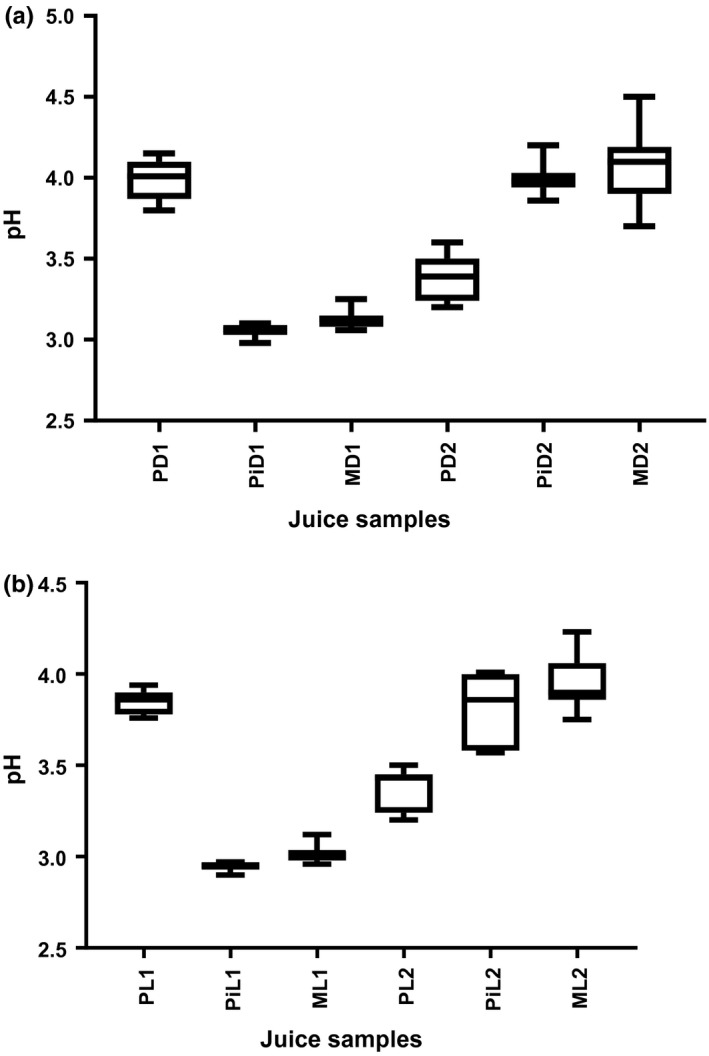
Mean pH values of juice samples stored in dark (amber)‐colored and clear (light) bottles at ambient (24°C) and refrigeration (4°C) temperatures. P‐Passion fruit, Pi‐ Pineapple, M‐mango, D‐ Dark bottle, L‐Light (clear) bottle, 1‐ Ambient (24°C) temperature, 2‐ Refrigeration (4°C) temperature

Overall, pH values decreased for all the samples over the 12‐day period (Figure 2). Values ranged from 3.87 to 3.80 for passion fruit, 3.05 to 3.03 for pineapple, 3.25 to 3.13 for mango juice samples; and 3.39 to 3.20 for passion fruit, 3.96 to 3.86 for pineapple, 4.19 to 3.90 for mango juice samples kept in dark bottles at ambient (24°C), and refrigeration (4°C) temperatures, respectively. (Figure 2a). pH values for juices stored in clear (light) bottles at ambient (24°C) and at refrigeration (4°C) temperatures were as follows: 3.82–3.76 for passion fruit, 2.96–2.94 for pineapple, 3.12–2.98 for mango juices and 3.43–3.20 for passion fruit, 4.01–3.75 for pineapple, and 4.06–3.86 for mango juices, respectively (Figure 2b). pH values for passion fruit in dark and clear bottles at ambient (24°C) temperature were within the range of 3.4–4.1 reported by Rocha and Bolini (2015). Tortoe et al. (2013) reported a pH value of 3.5 for pineapple (smooth cayenne), while Silva et al. (2015) reported a pH value of 3.86 for pineapple pulp which was within a similar range to pH for juice sample kept in dark bottle at refrigeration (4°C) temperature.

pH values for samples kept at ambient (24°C) temperature were significantly different (*p* < .05) from samples held at refrigeration (4°C) temperature for all samples in dark (amber) and clear (light) glass bottles. This was probably due to the presence of mesophilic bacteria such as *Staphylococcus aureus, Salmonella spp,* and *Escherichia coli* which acted on the nutrients like sugars in the samples resulting in production of organic acids, hence the low pH. Low pH favors the growth of acid‐tolerant bacteria leading to spoilage of the samples and shortened storage stability of the products. The data obtained in this study were higher than pH values (2.72–3.17) and (2.82) reported by Fernandes et al. (2011) and Silva et al. (2015), respectively. Janzantti, Santos, and Monteiro (2012) reported a pH value of 2.6 for fresh yellow passion fruit pulp. The difference in pH could probably be due to difference in varieties of the fruits used. The yellow passion fruit is the third most produced juice on the Brazilian market and is widely consumed for its high aroma and acidity (Fernandes et al., 2011), while the purple passion fruit is less acidic and is preferred for its aroma and flavor in Ugandan juices (Nyanzi, Carstensen, & Schwack, 2005). pH influences the type of microorganisms that will grow and survive in the juice and therefore stability of the juice. It also influences the stability of bioactive compounds in the juice (Chia, Rosnah, Noranizan, & Ramli, 2012).

Low pH generally tends to inhibit bacterial growth in fresh unpasteurized fruit juices (Nwachukwu and Ezeigbo, 2013), allowing acid‐tolerant pathogenic bacteria including *Salmonella* spp, *Staphylococcus aureus, and Listeria monocytogenes* to survive in the juice (Alonzo, 2009). *Escherichia coli* 0157:H7, *Salmonella* spp, and *Listeria monocytogenes,* exhibited homologous and heterologous adaptations to subsequent acid and thermal stresses (Alonzo et al., 2009; Aneja, Dhiman, Aggarwal, Kumar, & Kaur, 2014). Pathogens were able to survive in acidic environment of juices because they were able to regulate their internal pH at neutral pH using active and passive homeostasis (Aneja et al., 2014). Enteric bacteria induce enzymes that raise internal pH and activate enzymes involved in the protection and repair of proteins and DNA (Aneja et al., 2014). These adaptive mechanisms present in the microorganisms allow increase in number resulting in depletion of nutrients and possibly development of spores and toxins. High numbers may probably lead to accumulation of metabolic by‐products causing deterioration and spoilage of the juices, hence decreased storage stability.

**Figure 3:**
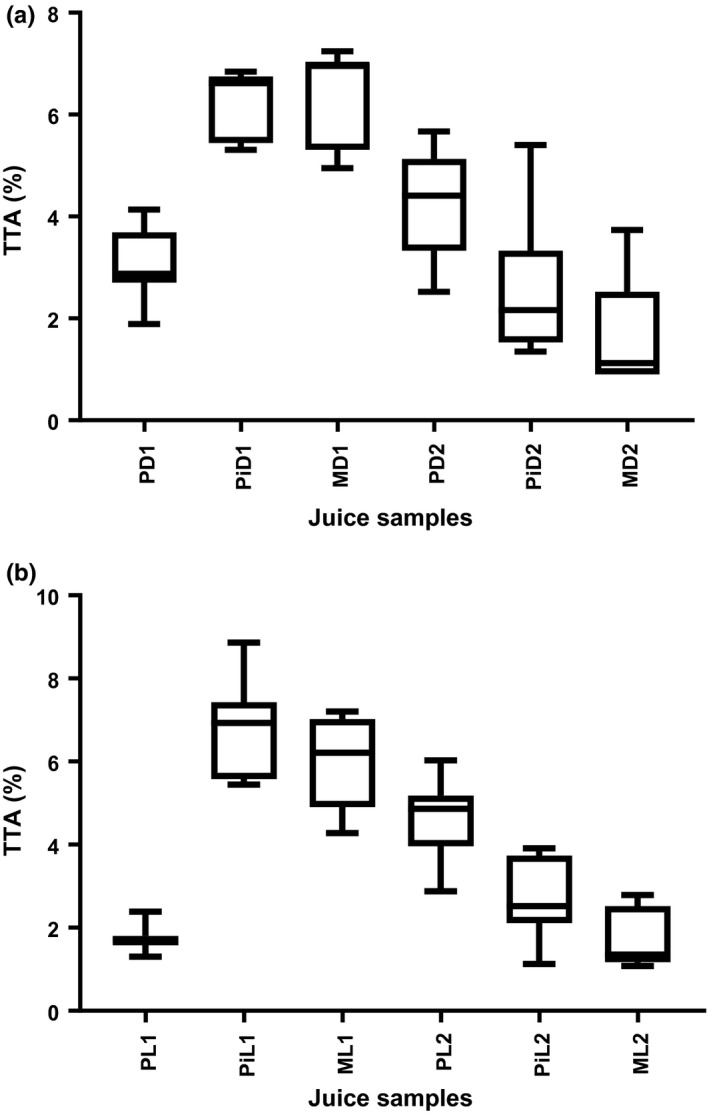
Mean values for TTA (%) of fresh juices kept in dark (amber)‐colored and clear (light) bottles at ambient (24°C) and refrigeration (4°C) temperatures. P‐Passion fruit, Pi‐ Pineapple, M‐mango, D‐ Dark bottle, L‐Light (clear) bottle, 1‐ Ambient temperature (24°C), 2‐ Refrigeration (4°C) temperature

Titratable acidity (TTA), the amount of acid in a food sample neutralized by a strong base was measured in the juice samples. Overall, TTA values for all juice samples decreased (Figure 3). Values obtained in the study ranged from 6.8 to 1.1 for juices in dark bottles stored at ambient (24°C) and at refrigeration (4°C) temperatures. TTA (%) values for samples kept in light bottles ranged from 7.0 to 1.3. The decrease in TTA (%) could probably have been due to the accumulation of organic acids from sugars produced by microorganisms' metabolic activities. The organic acids favor the development of microorganisms like yeasts in juices leading to a reduction in sugar content and fermentation leading to spoilage (Abbo et al., 2006). They reported TTA (%) values of 0.2 g citric acid/ 100 g and 0.07 g citric acid/ 100 g for juice stored at 4°C and 28°C by the eighth week, respectively (Abbo et al., 2006). Mango juice samples kept at ambient (24°C) temperature were significantly different (*p* < .05) from those kept at refrigeration (4°C) temperatures.

Samples kept in light bottles at ambient (24°C) temperature presented the following results: 1.3–1.8 for passion fruit, 5.4–7.4 for pineapple, 4.3–7.0 for mango; and 4.0–4.9 for passion fruit, 1.1–3.7 for pineapple, 1.6–2.8 mango juice samples. Samples kept at ambient (24°C) temperature were significantly different from those kept at refrigeration (4°C) temperature. Silva et al. (2015) reported TTA (%) values of 3.24 ± 0.01, 0.47 ± 0.02, 0.39 ± 0.04 for passion fruit, pineapple, and mango fruit pulps, respectively. The value reported by Silva et al. (2015) for passion fruit juice samples kept in dark (amber)‐colored bottles was within the range obtained in the study. Values for pineapple and mango juice samples in the study were higher than those mentioned by Silva et al. (2015) possibly because those they reported were for fresh pulps.

**Figure 4:**
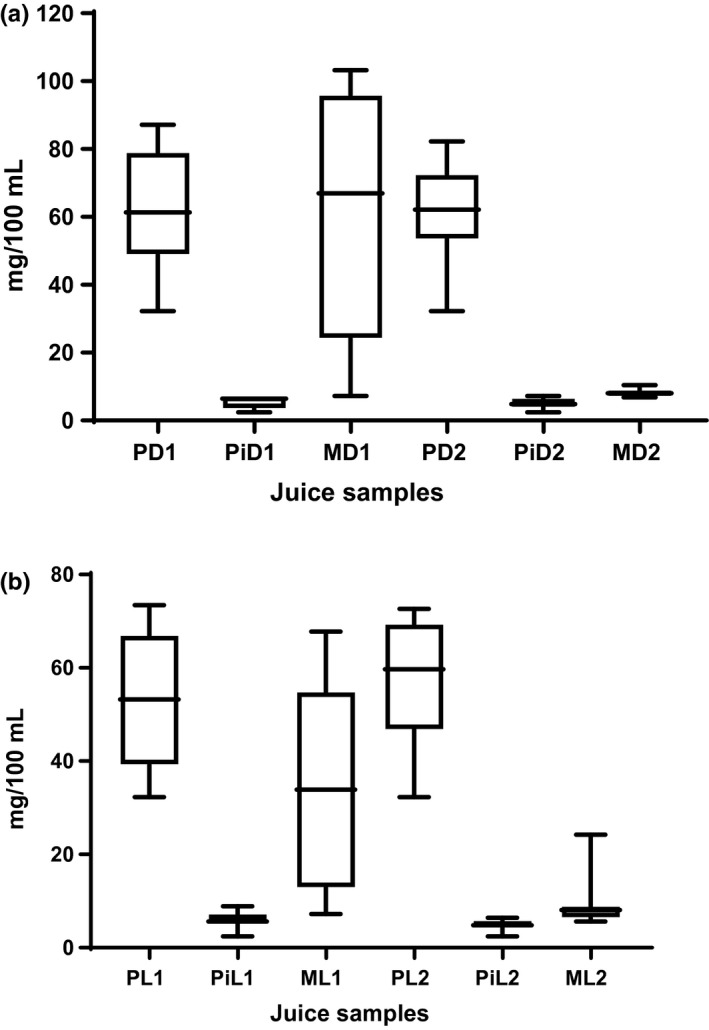
Mean ascorbic acid values of fresh unpasteurized fruit juices kept at ambient (24°C) temperature and at refrigeration (4°C) temperature. P‐Passion fruit, Pi‐ Pineapple, M‐mango, D‐ Dark bottle, L‐Light (clear) bottle, 1‐ Ambient (24°C) temperature, 2‐ Refrigeration (4°C) temperature

The ascorbic acid content increased gradually by the fourth day for passion fruit and mango juice stored at ambient (24°C) temperature; and thereafter steadily declined (Figure 4). Highest mean vitamin C content was observed in passion fruit juice samples kept at ambient (24°C) temperature (53.2–55.5 mg/100 ml) and at refrigeration (4°C) temperature (61.0 mg/100 ml). Lowest vitamin C content was observed in the pineapple for juice samples kept at ambient (24°C) temperature (4.7–5.0 mg/100 ml) and at refrigeration (5.9–6.0 mg/100 ml) temperature. Passion fruit and pineapple samples in dark (amber) glass bottles at ambient (24°C) temperature were not significantly different (*p* > .05) from samples in clear (light) bottles kept at refrigeration (4°C) temperature. This may probably be due to oxidation of ascorbic acid to dehydroascorbic acid, both of which display vitamin C biological activity, thus the probable increase by the fourth day (Bender, 2009). Pineapple juice stored in the dark‐colored and clear (light) bottles was fairly constant throughout the storage period. pH is the main factor affecting the stability of vitamin C; thus, high values of pH may be favoring the oxidation processes of vitamin C.

The maximum ascorbic acid content was observed in passion fruit juice for both the clear (light) and dark‐colored bottles. Mean values of ascorbic acid obtained for passion fruit juice were higher than those recommended by Bender (2009), which may probably be a suitable source of ascorbic acid for adults and the juice‐consuming population. An ascorbic acid daily intake of 60.0 mg is recommended for adults (Bender, 2009). The values obtained for passion fruit of 61.9 mg per 100 ml probably suggest that passion fruit juice in Kampala may provide a good and sufficient source of ascorbic acid for the consuming population. A value of 61.9 mg per 100 ml similar to the results obtained for passion fruit stored in the dark‐colored bottles was reported by De Sousa, Maia, De Azeredo, Ramos, and de Figueiredo (2010). Values obtained for ascorbic acid in this study were much higher than those reported by Genovese, Pinto, and Schmidt (2008) and Janzantti et al. (2012) for frozen pulp (4.3 mg/100 g) and (15.0 mg/100 ml) for fresh pulp in Brazil. This may probably be due to differences in the varieties of passion fruit used and regional differences under which the fruits are grown. Lowest ascorbic acid content (4.7–6.0 mg/100 ml) was observed in pineapple juice sample. Silva et al. (2015) reported ascorbic acid values of 21.43 ± 1.61, 19.86 ± 4.00, and 28.68 ± 3.55 mg/100 g for passion fruit, pineapple, and mango pulps. Vitamin C is usually oxidized first before all other bioactive components. When it is still present in sufficient quantities, it probably implies that other nutrients are still present indicating longer shelf life of the juice samples.

**Figure 5:**
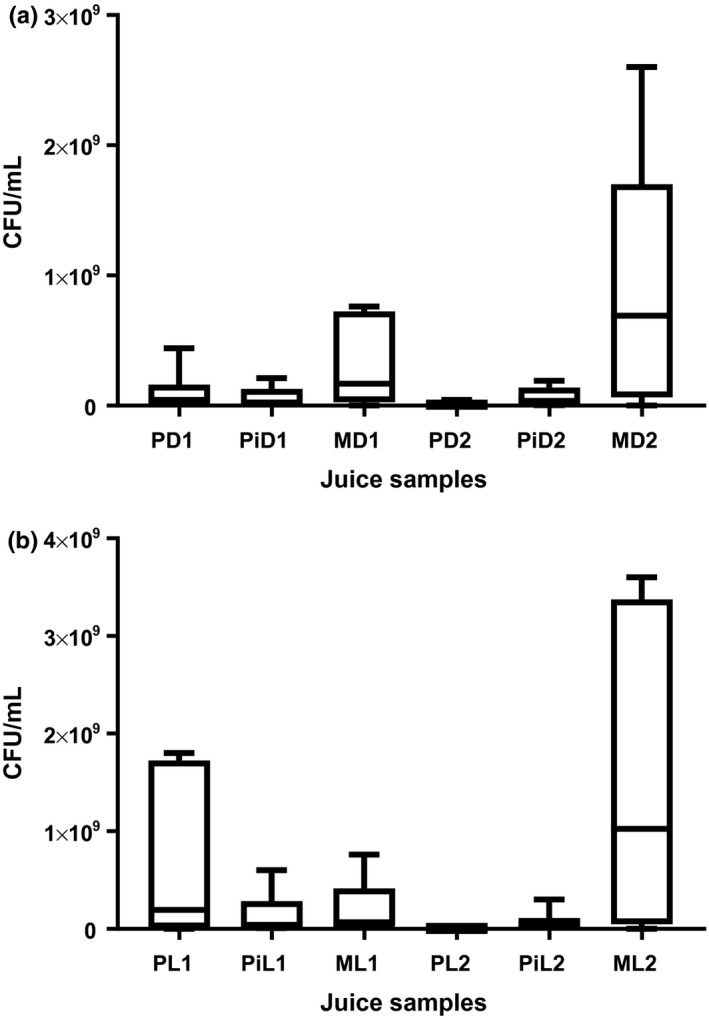
Mean total plate counts for fresh juices in dark (amber)‐colored and clear (light) glass bottles kept at ambient (24°C) and at refrigeration (4°C) temperatures. P‐Passion fruit, Pi‐ Pineapple, M‐mango, D‐ Dark bottle, L‐Light (clear) bottle, 1‐ Ambient (24°C) temperature (24°C), 2‐ Refrigeration (4°C) temperature

Generally, total plate count values for the juice samples increased over the days of storage (Figure 5). High mean total plate counts (TPCS) above the permitted levels of 10___ CFU/ml according to Codex Alimentarius Commission (CAC) of the Food and Agricultural Organization, (2003) were observed in all juice samples. Highest viable mean total plate count (1.5 × 10___ CFU/ml) was observed for mango juice stored at ambient (24°C) temperature in clear (light) bottle. Lowest mean value (3.9 × 10___ CFU/ml) was observed in passion fruit juice stored at ambient (24°C) temperature in clear (light) bottles. Values ranged from 5.4 × 10___ to 3.7 × 10___ CFU/ml for passion fruit, 1.3 × 10___–2.4 × 10___ CFU/ml for pineapple, 1.5 × 10___ CFU/ml for juices kept in dark (amber)‐colored bottles at ambient (24°C) temperature and 3.5 × 10³–2.9 × 10___°CFU/ml for passion fruit, 7.6 × 10___–2.8 × 10___°CFU/ml for pineapple, and 1.2 × 10___–1.8 × 10___°CFU/ml for juices in dark (amber)‐colored glass bottles kept at refrigeration (4°C) temperature.

For juice samples in light bottles at ambient (24°C) temperature, ranges for total viable plate counts were as follows: 1.8 × 10___ CFU/ml for passion fruit, 2.4 × 10___–8.0 × 10___ CFU/ml for pineapple, 1.7 × 10___–4.8 × 10___ CFU/ml for mango; and 2.7 × 10___–2.6 × 10___ CFU/ml for passion fruit, 1.3 × 10___–7.0 × 10___ CFU/ml for pineapple, and 1.2 × 10___–1.5 × 10___ CFU/ml for mango juice samples kept at refrigeration (4°C) temperature. Values of 2.94 × 10___–3.46 × 10___ CFU/ml for apple juice, 2.90 × 10___–3.30 × 10___ CFU/ml for orange juice, 2.82 × 10___–3.26 × 10___ CFU/ml for pineapple juice, and 3.43 × 10³ CFU/ml for mango juice have been reported by Nayik et al. (2013). Values obtained in this study were higher than those reported by Nayik et al. (2013) possibly due to the presence of epiphytic microflora on the surface of the fruits. These may find their way into the juice when the fruits are cut during extraction (Baghurst, Beaumont‐smith, Natalie Baghurst, & Cox, 1999; Farber et al., 2003; Centre for food safety fruits for sale or serving in retail outlets, 2006; Artes & Allende, 2012). Breidt and Caldwell (2011) also noted the presence of microflora on fresh fruits, grains, and vegetables ranging from 10² to 10___ colony‐forming units (CFU) per gram.

Microorganisms are introduced into juices during minimal processing when fruits and vegetables are cut, sliced, skinned, or shredded, which removes or damages the protective surfaces of the fruit (European Commission, 2002; Artes & Allende, 2012). Poor hygiene during processing and handling of the juices could also have contributed to the high total plate counts compromising the safety of these products. This in turn could put the consuming population at risk of contracting foodborne illnesses in addition to potential wastage as a result of spoilage. Nwachukwu and Ezeigbo, (2013) also observed a high microbial load of TPC 4.3 × 10___ CFU/ml on first day of storage to 28.6 × 10___ CFU/ml on the 14th day of storage for soursop juice.

Fruit and vegetable juices may also be further contaminated with fecal pathogens from pickers and handlers during harvesting, and packaging equipment, as well as dust and dirt or even dirty water from surface runoff when the products are offloaded onto the ground on arrival to the market (European Commission, 2002). The low total plate counts for ambient (24°C) temperature passion fruit juice samples for clear and dark‐colored bottles could probably be due to reduced pH resulting from bacterial metabolic activities. The metabolic activities could possibly have resulted in biodeterioration of juice samples reducing shelf life of the products.

## 4 CONCLUSION

Hygienically processed unpasteurized juice stored at ambient (24°C) and refrigeration (4°C) temperatures may remain safe for consumption for 24 and 48 hr, respectively. Physicochemical parameters of fresh unpasteurized juices significantly changed affecting storage stability. pH, Brix, titratable acidity (%)—all decreased probably due to the high microbial load, resulting in biodeterioration, spoilage, and shortened storage stability of juices stored both in dark (amber)‐colored and clear (light) glass bottles at ambient (24°C) and at refrigeration (4°C) temperature. Fruit juices need to be protected from factors like temperature, especially during handling and preparation of food products that accelerate changes in physicochemical parameters resulting in biodeterioration and spoilage of the products. Ascorbic acid content remained high indicating the possibility of food products such as fruit juices being used to supply adequate amounts to satisfy adult daily human requirements. High microbial load indicates probable contamination from unhygienic processing methods and poor personal hygiene reflecting poor microbiological quality of fresh unpasteurized fruit juices.

## CONFLICT OF INTEREST

None declared.
